# Comparison of the Long-Term Efficacy of Targeting the Subthalamic Nucleus Versus the Globus Pallidus Interna for Deep Brain Stimulation Treatment of Motor Dysfunction in Patients With Parkinson's Disease: A Meta-Analysis Study

**DOI:** 10.1155/padi/5157873

**Published:** 2024-11-26

**Authors:** Makenna Huhn, Matthew Prewett, Julien Rossignol, Gary L. Dunbar

**Affiliations:** ^1^Program in Neuroscience, Central Michigan University, Mount Pleasant 48859, Michigan, USA; ^2^College of Medicine, Central Michigan University, Mount Pleasant 48859, Michigan, USA; ^3^Department of Psychology, Central Michigan University, Mount Pleasant 48859, Michigan, USA

## Abstract

A cardinal symptom of Parkinson's disease (PD) is motor dysfunction, including bradykinesia and tremors, which is quantified in the Unified PD Rating Scale (UPDRS). Although some medications provide palliative treatments for these motor deficits, their efficacy wanes and can produce unwanted side effects, such as dyskinesia. Deep-brain stimulation (DBS) has provided an alternative treatment strategy that can benefit many patients, but optimal target structures for DBS and its long-term efficacy are not fully understood. The present study represents a meta-analysis of the long-term (> 5 years) effects of DBS on the two most common targets, the subthalamic nucleus (STN) and the globus pallidus interna (GPi), on scores of motor performance using the UPDRS-III. The initial search of PubMed, Cochrane Library, and Clinical Trials resulted in 197 articles, of which 28 met the criteria for our analysis. Of the 1321 patients included, 1179 received STN DBS group and 142 received GPi DBS. UPDRS-III scores for both target groups were analyzed at baseline and at either 5–8 or 10–15 years later for both on- and off-medication phases. The results indicated that the STN stimulation is effective at reducing motor symptoms during off-medication treatment for up to 15 years and that the GPi stimulation can be effective for up to at least 8 years. Our findings further suggest that STN- and GPi-targeted DBS may wear off during the on-medication phase between 5 and 10 years of treatment. This study supports findings that both DBSs of either the STN or GPi have long-term efficacy, especially during off-medication periods.

## 1. Introduction

Parkinson's disease (PD) is a progressive, neurodegenerative disease associated prominently with the gradual loss of dopamine-producing cells in the substantia nigra pars compacta (SNc) and concomitant dysfunction in the basal ganglia, or more appropriately, the basal nuclei (BN) of the brain [1]. Motor abnormalities, such as tremors, rigidity, bradykinesia, and balance issues, are manifested in PD, along with a host of other symptoms, including cognitive decline, depression, and gastrointestinal issues, which emerge throughout the progression of the disease [2]. Currently, there are no effective, long-term treatments for PD although the use of dopaminergic (DA) agonists, such as leva-dopamine (L-Dopa), and cell-replacement therapies have shown short-term palliative benefits in reducing some of the motor dysfunction [3]. However, the use of DA-enhancing medication is temporary and fraught with complications, such as L-Dopa-induced dyskinesia and psychotic symptoms [3]. Thus, it has become increasingly apparent that the etiology of PD symptoms is more complex than just the degeneration of the DA-producing cells in the SNc [4]. Despite our incomplete understanding of the nuances of PD pathology, manipulations of the BN circuitry, through lesions or deep-brain stimulation (DBS) of key BN structures, can facilitate the benefits of DA-enhancing medication and/or provide an alternative therapy for reducing PD symptoms.

Our understanding of the BN circuitry continues to evolve with new discoveries of the nuances of parallel pathways that help mediate motor, cognitive, and affective functions, along with reciprocal connections between several key structures, which obfuscate simple explanations of how the BN ultimately affects movement [5]. Nonetheless, clinical investigations into altering the functions of key BN structures, primarily the globus pallidus interna (GPi) and the subthalamic nucleus (STN), by lesions or DBS have proven to be relatively effective in reducing aberrant motor symptoms in some PD patients [6].

Most of the studies using lesions or DBS of the GPi or STN have been done in patients with advanced PD [7, 8], when medications, such as L-Dopa, lose their efficacy or produce significant side effects, such as L-Dopa-induced dyskinesia [6]. More recently, clinicians have focused on the use of DBS, rather than lesions of BN structures for treating PD, given that DBS allows for adjustments, such as voltage regulation, which is not possible after lesions of these areas [9]. The use of DBS can be effective in reducing motor symptoms during “off” periods of medication, and the coupling of medication and stimulation is often preferred over medication alone to optimize control of motor symptoms [10]. DBS requires the surgical implantation of thin electrodes into the target area of the brain, which connect to an impulse generator battery, or neurostimulator, which commonly is placed under the collarbone of the patient, allowing for remote control for optimizing voltage levels [11].

DBS targeting either the STN or the GPi can reduce motor symptoms in some PD patients [7] although results can vary between individuals, given that each target has its own peculiar benefits and side effects. For example, STN stimulation may be more efficient in lowering daily levodopa dosage [7]. However, reports of worsening depression symptoms after STN stimulation suggest that GPi stimulation may be preferable for PD patients who are comorbid with depression [12]. Although some studies suggest that the use of the STN-targeted DBS may be more efficacious, at least during the “off-medication” periods [13–15], there is some evidence that GPi-targeted DBS may be superior to STN-targeted DBS in reducing L-DOPA-induced dyskinesia [16]. A recent study from Hwang and colleagues [17] reports stimulation in the GPi may yield better results in the on-medication phase compared to STN, suggesting a need for more studies that directly compare the use of STN or GPi stimulation for subpopulations of PD patients [6].

The majority of recent studies have focused on STN-targeted DBS, as growing evidence supports its efficacy in reducing motor symptoms in elderly PD patients [18], reducing motor deficits as assessed by the Unified PD Rating Scale (UPDRS) by 45.7%. In addition, STN-targeted DBS reduced daily dosing of levodopa in most patients by 40%–50% [19, 20]. The use of STN-targeted DBS appears to be more effective during the “off” stage than during the “on” stage of levodopa medication [19].

Although fewer studies have used GPi-targeted DBS for treating PD, there is ample evidence that it can also reduce motor symptoms [7, 15, 21, 22]. Targeting GPi seems to have a particular advantage in treating dyskinesia [10, 15, 19]. Although GPi-targeted DBS works well during the “on” stage of levodopa medication [17], evidence for its ability to reduce daily dosing of levodopa is lacking [7, 19, 20].

Studies that directly compare the efficacy of STN- and GPi-targeted DBS for PD have provided interesting insights. The use of STN DBS has been reported to decrease action and postural tremors more than GPi DBS at 6 months, but not 12 months postoperatively [23]. In 2021, Zhang and colleagues [15] conducted a meta-analysis comparing the efficacy of STN-targeted and GPi-targeted DBS for treating motor symptoms in PD patients. They concluded that both STN and GPi were equally effective as targets for DBS as a means of reducing motor dysfunction. However, stimulation of the STN was more efficacious in reducing the need for medication, while targeting the GPi for DBS stimulation was more effective in counteracting deficits in essential activities of daily living (ADL) and in reducing dyskinesia when the patient was on medication [15]. Correspondingly, Peng and colleagues [14] have shown that GPi DBS can improve daily living in patients during both the “on” and “off” medication phases. On the other hand, Odekerken and colleagues [24] have shown that STN DBS reduced off-drug phase motor deficits more than GPi DBS, without affecting quality of living scores at 3 years postoperatively. Although this study indicated that this effect was apparent within the first 3 years following the DBS, the longer-term effects of STN- or GPi-targeted DBS on reducing motor dysfunction need further exploration.

Unfortunately, most of the studies comparing STN- and GPi-targeted DBS treatments for PD used relatively short-term follow-ups, usually under 5 years. Less is known about the long-term consequences of using either STN or GPi as targets for DBS therapy in PD. In this context, the present study attempted to utilize a meta-analysis approach to compare the efficacy of STN and GPi as targets for treating PD utilizing longer-term follow-ups of 5 years or more.

## 2. Methods

### 2.1. Literature Search and Study Selection

We conducted an extensive search using PubMed, Cochrane Library, and Clinical trials up to October 31, 2023. The keywords used to search for studies utilizing DBS in PD patients to evaluate motor symptoms following UPDRS Part III criteria were “subthalamic nucleus OR STN,” “globus pallidus OR GPi,” “deep brain stimulation OR DBS,” “parkinsons OR parkinson disease OR parkinson's disease OR parkinson's,” “UPDRS,” and “long-term.”

Titles, abstracts, and full texts were evaluated by the authors according to the following inclusion criteria: (1) unilateral or bilateral DBS of STN or GPi, using either comparative study or single target study; (2) DBS as the main intervention/treatment; (3) UPDRS-III on- and/or off-medication scores used as measurement for motor symptoms; (4) patient follow-up of at least 5 years; (5) inclusion of control group of patients at baseline or pre-DBS; and (6) study method included either randomized controlled trial, prospective cohort study, or retrospective cohort study.

Studies were excluded if they (1) only assessed short-term results (< 5 years); (2) studied pathologies other than PD; (3) compared STN or GPi to a different stimulation site (e.g., ventral intermediate nucleus) or different treatment (e.g., best medical treatment); (4) did not report data in means and standard deviation (SD) or standard error (which would be converted to SD), and (5) were not published or translated in English.

Some search results included studies derived from previous studies found during the search, for example, a published study building off an original study by extending the follow-up period. In these cases, the study with the highest number of participants and longest follow-up period was included in this analysis. In addition, multiple studies only reported data in the on-medication phase or off-medication phase, not both. These studies were included and only used in the analyses for which they could contribute useful data.

### 2.2. Data Extraction

The data extracted from the studies included the study design, number of patients in each DBS target, average age of the patient at the time of baseline measurements, disease duration of the patient at the time of baseline measurements, male-to-female ratio, trial duration, and UPDRS-III scores for on- and off-medication phases. The UPDRS was used to assess motor, functional, and cognitive symptoms of PD patients. The higher the score, the more severe the symptoms. Part 3 out of 4 parts of the UPDRS measures the motor function, which is the focus of this study.

All studies meeting the inclusion criteria reported means and SD of baseline and postoperative results.

### 2.3. Statistical Analysis

Since all measurements were continuous data, we pooled data by calculating Hedges' *g* standard mean difference between the baseline scores and specific follow-up period scores. The pooled data were analyzed using the random effect inverse variance method of DerSimonian and Laird [25]. This method uses the variance of each study to assess its weighted contribution to the analysis. The results were reported in 95% confidence intervals, *z* scores, and *p* values. The *Q* and *I*^2^ statistics were computed to test the heterogeneity of effect sizes between studies. Different *I*^2^ values indicate levels of heterogeneity, those being 25% for low, 50% for moderate, and 75% for high heterogeneity. We used a random effects model if high heterogeneity existed in each analysis. The data calculations and analyses were performed using R Statistical Software v4.3.2 [26] and the metafor R package v4.4.0 [27].

## 3. Results

### 3.1. Eligible Studies and Patient Characteristics

The initial search of PubMed, Cochrane Library, and Clinical Trials resulted in 197 articles. After sorting through titles and abstracts, 53 full-text studies were reviewed for eligibility and duplicates, yielding 28 accepted studies. A total of 1321 patients were included, with 1179 in the STN DBS group and 142 in the GPi DBS group. The average age of all participants was 57.87 years old, and the average age across each study ranged from 43.8 to 64.9. The average age of the STN group was 57.46 years old, and the average age of the GPi group was 60.33 years old. Moreover, the average duration of disease for all patients was 11.79 years, with the average for STN and GPi groups being 11.62 and 13.37, respectively. UPDRS-III total scores were measured at baseline for both on- and off-medication phases. The average score for the on-medication phase was 20.23 for the STN group and 22.58 for the GPi group. The average score for the off-medication phase was 48.07 for the STN group and 45.47 for the GPi group. Follow-up periods ranged from 5 to 15 years. The characteristics at the baseline of each group are listed in Table 1.

A complete list of the studies used in the meta-analysis, including the number of patients, their ages, gender, surgical procedure, outcome measure, duration of the disease, and follow-up interventions are listed for both the STN-targeted DBS studies and GPi-targeted DBS studies in Table 2.

### 3.2. Overall Motor Outcomes

Motor symptoms were measured using the UPDRS-III in two different phases: off-medication/on-stimulation (off phase) and on-medication/on-stimulation phase (on phase).

**Figure 1:**
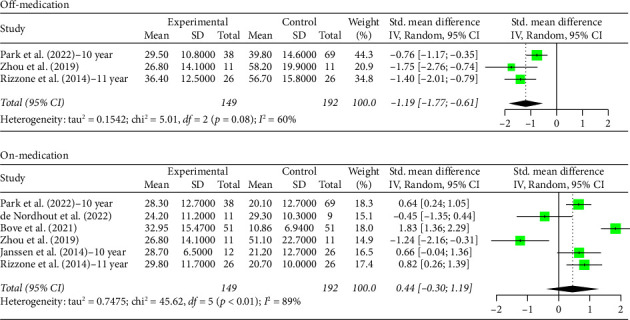
Forest plot: meta-analysis of standardized mean differences for all studies of STN DBS treatment and control in PD treatment during off- and on-medication treatment. CI, confidence interval; SD, standard deviation.

**Figure 2:**
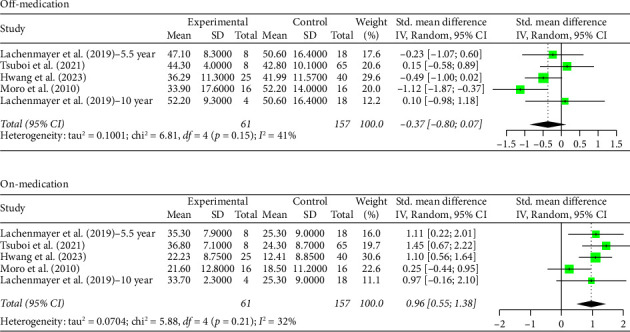
Forest plot: meta-analysis of standardized mean differences for all studies of GPi DBS treatment and control in PD treatment during off- and on-medication treatment. CI, confidence interval; SD, standard deviation.

The STN group had a statistically significant decrease in motor scores from baseline (off-stimulation) in the off-medication phase across all follow-up periods, with Hedges' *g* = −1.5821 (95% CI [−1.9956; −1.1686], *p* < 0.0001, *I*^2^ = 92.6%; Figure 1). In the on-medication phase, STN group scores increased from baseline, Hedges' *g* = 0.5134 (95% CI [0.3014; 0.7254], *p* < 0.0001, *I*^2^ = 78.1%; Figure 1). The off-medication phase for the GPi group across all follow-up periods indicated a nearly significant trend toward a decrease in motor scores, while the on-medication phase indicated a statistically significant increase in motor scores. Hedges' *g* for the off-medication phase had a value of −0.3675 (95% CI [−0.8037; 0.0687], *p*=0.0987, *I*^2^ = 41.2%; Figure 2) and a value of 0.9638 (95% CI [0.5504; 1.3773], *p* < 0.0001, *I*^2^ = 32.0%; Figure 2) for the on-medication phase. The *I*^2^ values of > 75% indicate high heterogeneity in each analysis, which warrants smaller subgroup analyses as high heterogeneity suggests that there is variability in our results outside of the accounted effect sizes. We broke up data according to follow-up periods, as studies with 10 or more years of follow-up also reported follow-up scores at 5 or more years. Separate analyses were conducted by grouping studies with data reported at 5–8 years follow-up and 10 or more years follow-up.

Two outliers exist in the STN off-medication analysis: Lezcano et al. [28] and Li et al. [29]. A sensitivity analysis shows no significant change in the results when the analysis was done either with or without these outliers (SMD = −1.5821 and SMD = −1.4656, respectively). One outlier existed in the STN on-medication analysis: Bove et al. [22]. A sensitivity analysis revealed no significant change in the results when the analysis was done either with or without the outlier (SMD = 0.5134 and SMD = 0.4653, respectively). Therefore, the results remain robust. No outliers were detected for the GPi off- and on-medication groups.

### 3.3. 5+ Year Analysis

**Figure 3:**
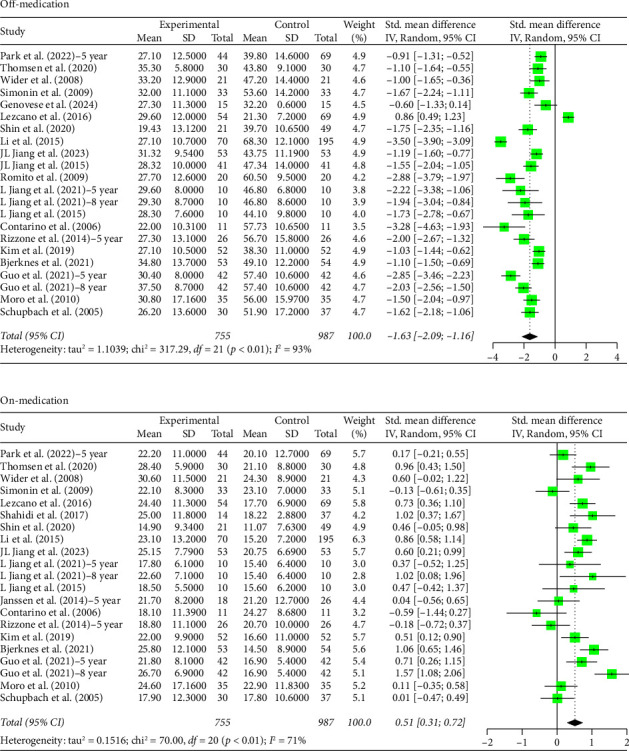
Forest plot: meta-analysis of standardized mean differences for STN DBS treatment and control in PD treatment at 5–8 years of follow-up during off- and on-medication treatment. CI, confidence interval; SD, standard deviation.

**Figure 4:**
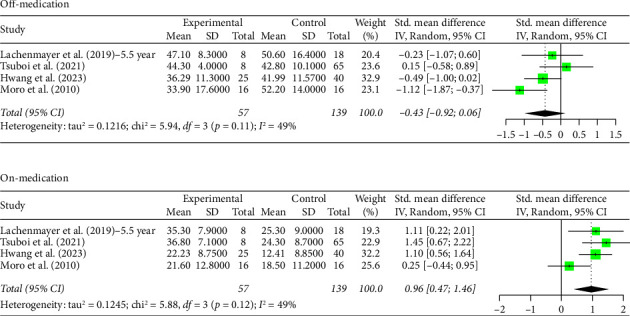
Forest plot: meta-analysis of standardized mean differences for GPi DBS treatment and control in PD treatment at 5–8 years of follow-up during off- and on-medication treatment. CI, confidence interval; SD, standard deviation.

The number of years patients underwent stimulation in this subgroup ranged from 5 to 8 years. Analysis of the group with STN stimulation yielded similar results to the original group for both off and on phases. Hedges' *g* for the off phase had a value of −1.6268 (95% CI [−2.0895; −1.1640], *p* < 0.0001, *I*^2^ = 93.4%; Figure 3), and Hedges' *g* for the on phase had a value of 0.5121 (95% CI [0.3079; 0.7162], *p* < 0.0001, *I*^2^ = 71.4%; Figure 3). The GPi group had a decrease in scores in both the off- and on-medication phases, with a Hedges' *g* value of −0.4315 (95% CI [−0.9194; 0.0564], *p*=0.0831, *I*^2^ = 49.5%; Figure 4) and 0.9649 (95% CI [0.4683; 1.4614], *p*=0.0001, *I*^2^ = 49.0%; Figure 4), respectively. High heterogeneity was observed in the off phase of the STN group. Moderate heterogeneity was observed in the on phase of the STN group and both phases of the GPi group.

### 3.4. 10+ Year Analysis

**Figure 5:**
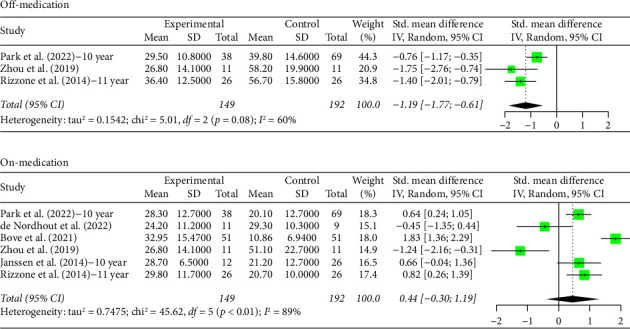
Forest plot: meta-analysis of standardized mean differences for STN DBS treatment and control in PD treatment at 10–15 years of follow-up during off- and on-medication treatment. CI, confidence interval; SD, standard deviation.

The number of years patients underwent stimulation in this subgroup ranged from 10 to 15 years. Analysis of the patients in the STN off medication group revealed a significant decrease in scores from baseline, Hedges' *g* = −1.1928 (95% CI [−1.7730; −0.6126], *p* < 0.0001, *I*^2^ = 60.1%; Figure 5), but with an increase in scores from baseline in the on phase, Hedges' *g* = 0.4445 (95% CI [−0.3000; 1.1890], *p*=0.2419, *I*^2^ = 89.0%; Figure 5). There was moderate heterogeneity in the off-phase group and high heterogeneity in the on-phase group.

Only one study recorded data for patient follow-up of 10 years with GPi stimulation. Hedges' *g* was calculated using the score at baseline and score at 10 years of stimulation and was found to be 0.0990 (95% CI [−0.9849; 1.1829], *p*=0.8579) for the off phase and 0.9683 (95% CI [−0.1598; 2.0963], *p*=0.0925) for the on phase, indicating that motor scores increased for both groups with no statistical significance. Interestingly, the scores for this group tended to increase, while the scores for the 5-year GPi group increased.

## 4. Discussion

To our knowledge, this meta-analysis is the first to compare the long-term efficacy of STN- and GPi-targeted DBS as a treatment for motor symptoms in PD patients with follow-up periods of 5 to 10+ years. This study reported the comparisons of UPDRS-III motor scores between the two targets and attempted to establish a target preference based on long-term motor outcomes.

Using our inclusion criteria, 24 of the studies reviewed utilized the STN as the target for DBS, with only three focused on the GPi (*n* = 3) and one study which included data on both target locations. Interestingly, an earlier study by Okun and Foote [30] predicted that GPi stimulation would be a preferred target for DBS because of fewer adverse postoperative effects when compared to the STN stimulation, but this did not materialize.

Multiple meta-analyses since the study by Kleiner-Fisman and colleagues [31] have reported positive improvement in motor symptoms after STN and GPi DBS, with STN DBS showing greater potential improvement than targeting the GPi [14]. The results of our study tend to support this conclusion and extend it to include a longer-term follow-up. Our findings suggest that both STN and GPi DBS improve long-term motor functions in PD patients during off-medication states, but not during on-medication states. Earlier studies by Peng and colleagues [14] and Zhang and colleagues [15] report that DBS therapy for both target areas is efficacious in the on- and off-medication phases for up to 3 years. However, our findings suggest that any beneficial effects of DBS therapy in the on-medication state diminish by 5 years. We analyzed the patient data into two treatment duration groups, one receiving 5–8 years of stimulation and the other 10+ years of DBS. Patients in the 5–8-year group showed significant motor improvement scores in the off-medication stage, but not in the on-medication stage, regardless of whether the site of stimulation was the STN or GPi, a finding which is consistent with previous studies [15]. Unfortunately, direct comparisons between STN and GPi as targeted sites for DBS in PD patients at 10+ year follow-ups could not be made because there was only one study that utilized GPi in this subgroup. Nonetheless, our findings did confirm those of others [32] who found significant long-term preservation of motor function in patients receiving STN stimulation during the off-medication phase.

Although the primary focus of our analyses was on comparing the two major targets for DBS on motor functioning, the effects on nonmotor functioning should not be ignored. Many studies support the use of STN-targeted DBS for lowering levodopa doses in patients with PD, while the use of GPi-targeted DBS does not typically lead to a reduction in levodopa dosage [7, 19, 20]. Because levodopa dosages can be lowered in patients with given STN-targeted DBS, it is reasonable to expect that the dyskinesia in these patients may be mitigated. On the other hand, GPi has been shown to be particularly effective at reducing dyskinesia symptoms and activities of daily living within the first 5 years of treatment, and it may offer a means of improving the quality of life of the patient shortly after initiating DBS [15, 19, 25].

In addition, mood alterations of patients are often considered in treatment plans. Some studies suggest that elevated mood swings have been noted following STN DBS, including worsening of episodes of depression, which are reportedly much less common in patients receiving GPi-targeted DBS [12]. However, some recent reviews reveal new data that seem to be largely heterogeneous regardless of the DBS target [33, 34]. This variability may be due to the difficulty of interpreting depression and anxiety scores as they may be influenced by the anticipation of upcoming intervention treatment or improved mood upon improved motor symptoms posttreatment [33, 34]. One mood change that has been increasingly recognized is apathy. Multiple studies report higher apathy scores following STN DBS, specifically bilateral DBS [35, 36]. A recent meta-analysis supports these findings and suggests preliminary results that GPi DBS may affect apathy less than STN DBS in PD patients although the exact effect of GPi DBS on apathy is still inconclusive [37].

Executive function and inhibitory control are also important to consider when selecting a target for DBS. Multiple studies favor GPi DBS in the context of executive function [33, 34]. More specifically, patients with GPi stimulation had no significant change in scores on the Stroop test, which measures inhibitory control, and better attention and working memory than patients with STN stimulation [33, 38]. Interestingly, some reports suggest that unilateral stimulation might be preferable to bilateral stimulation, specifically with STN DBS, when it comes to executive function [33, 34, 38]. A caveat to these findings is that age is a predictor of a decline in executive function, as older patients are susceptible to cognitive decline. Therefore, age may confound DBS effects on cognitive function posttreatment, especially when evaluating follow-up times of 10+ years since DBS candidates are typically older.

Of particular note, DBS may significantly decrease verbal fluency. A meta-analysis by Elgebaly et al. [33] notes that multiple studies showed a decline in verbal fluency in the STN DBS group, but not in the GPi DBS group, but their pooled analysis failed to show a statistically significant difference between the two. This is consistent with another meta-analysis [34] and a focused study of 11 patients with STN DBS that showed a significant decline in phonemic and semantic fluency and had consistent results with other studies utilizing GPi DBS [39]. As of now, the literature seems to agree that verbal fluency decreases following DBS treatment, with no consistent, significant differences between STN and GPi DBS. Newer research is attempting to discern how different frequencies and the location of stimulation may improve these fluency side effects [40], but this is outside of the scope of this study. Clinicians should consult this literature when looking at treatment plans.

These findings suggest that the choice of using STN- or GPi-targeted DBS should take into consideration the psychological history of the patient as well as their response to levodopa medication. If both target areas have comparable effects on motor symptoms of PD for up to 8 years, then GPi may be the target of choice considering the lessened negative effects on mood and executive function symptoms and the ability to reduce med-induced dyskinesia.

Some limitations to this study, beyond the publication bias of reporting a disproportionate number of positive findings, include a relatively high degree of heterogeneity (as indicated by a relatively high *I*^2^) in the data due to methodological issues, limited patient data, and the lack of long-term studies using GPi-targeted DBS. Methodological factors such as the precise placement of stimulating electrodes and the stimulation frequency and pulse width undoubtedly contributed to some of the variabilities observed in the data. For example, slight deviations in the placement of electrodes may significantly affect therapeutic outcomes for either STN- or GPi-targeted DBS. In terms of electrode placement in the STN, the dorsolateral sensorimotor part is the optimal location for clinical efficacy in controlling motor function [41]. Significant variations in the anatomical structures between patients can alter the precise placement of the electrodes, which may limit the effectiveness of the DBS treatment. Another limitation may be the use of unilateral versus bilateral stimulation, which can affect the quality of living measures within the first 18 months postoperatively [42], but this did not appear to affect the long-term motor outcomes following GPi DBS [33]. Since only one study reported unilateral stimulation and the other studies did not specify whether the DBS was unilateral or bilateral, our analysis could not perform a subgroup analysis on stimulation type. Limited patient data prevented analyses of different ethnic groups, which likely contributed to the variability observed. Some studies had data collected exclusively from Chinese patients, and the limited sample sizes precluded analyses based on differences in lifestyle and genetic backgrounds, which could have influenced some of the outcomes. In addition, only a few studies used GPi-targeted DBS, which precluded comparisons of efficacy between the target sites at the 10+ year follow-ups. Therefore, STN-targeted DBS data were compared to one study of GPi-targeted data, and caution should be taken when interpreting this result. In addition, not every study reported the off-medication or on-medication motor scores, reducing the power of our analyses. Finally, the statistical comparisons were limited to motor symptoms, so only qualitative comparisons between DBS targets on cognitive and executive functioning in PD patients were provided in this study.

## 5. Conclusions

This study provides confirmation that STN stimulation is effective at reducing motor symptoms during off-medication treatment for up to 15 years, and GPi stimulation can be effective for up to 8 years. Our findings further suggest that STN- and GPi-targeted DBS may wear off during the on-medication phase between 5 to 10 years of treatment. When considering the nonmotor symptoms, GPi DBS may be the target of choice. In several nonmotor categories, including apathy and executive control, the negative effects of GPi DBS are not worse and sometimes less than those following STN DBS. The present study indicates that further research comparing the long-term efficacies of STN- and GPi-targeted DBS is warranted as the selection of the DBS target may depend on several factors, including the medical and psychological history of the patient, anticipated duration of levodopa treatments, and specific location of electrodes in the target area. Therefore, personalized treatment plans for patients are still encouraged and important to consider.

## Figures and Tables

**Table 1:** Patient characteristics of the included studies.

Targeted structure	STN	GPi
Number of participants	1179	142
Mean age of participants	57.46	60.33
Duration of the disease (years)	11.62	13.38
Gender of participants	57.7% male; 42.3% female	59.7% male; 40.3% female

**Table 2:** Overview of articles included STN studies.

Study	No. of patients	Average age	Male	Female	Intervention	Duration of intervention	Average duration of disease
Park et al. [43]	69	61.7	37	44	Bilateral STN DBS	5 and 10	11.3
Thomsen et al. [44]	30	56.1	19	11	Bilateral STN DBS	8 to 15	11.5
Wider et al. [45]	21	64.9	30	20	Bilateral STN DBS	5	14.4
Simonin et al. [46]	33	NR	NR	NR	Bilateral STN DBS	5	NR
Genovese et al. [47]	15	57.1	8	7	Bilateral STN DBS	5	12.9
Lezcano et al. [28]	69	61.1	31	23	Bilateral STN DBS	5	13
Shahidi et al. [48]	37	50	26	10	STN DBS∗	6	11.3
Shin et al. [49]	49	59.5	18	31	Bilateral STN DBS	5	15.9
Li et al. [29]	195	58.2	105	90	Bilateral STN DBS	5	6.8
Jiang et al. [50]	53	58.89	24	29	Bilateral STN DBS	5	9.36
Jiang et al. [51]	41	56.3	29	12	Bilateral STN DBS	5	10.2
Romito et al. [52]	20	56.4	NR	NR	Bilateral STN DBS	5	14.3
Jiang et al. [53]	10	55.4	7	3	Bilateral STN DBS	5 and 8	8.9
Jiang et al. [54]	10	59.4	6	4	Bilateral STN DBS	5	9.3
Janssen et al. [55]	26	58	18	8	Bilateral STN DBS	5 and 10	12.7
Contarino et al. [56]	11	57.5	7	4	Bilateral STN DBS	5 and 10	15.5
Rizzone et al. [57]	26	57.8	18	8	Bilateral STN DBS	5 and 11	15.3
Kim et al. [58]	52	58.7	27	25	Bilateral STN DBS	5 to 7	12.5
Bjerknes et al. [59]	54	63	39	15	STN DBS∗	5	12
Guo et al. [60]	42	56.6	27	15	Bilateral STN DBS	5 and 8	6.8
Moro et al. [61]	35	59.6	17	18	Bilateral STN DBS	5 to 6	14.1
Schupbach et al. [62]	37	54.9	24	13	Bilateral STN DBS	5	15.2
de Noordhout et al. [63]	9	63	NR	NR	Bilateral STN DBS	12	9.4
Bove et al. [22]	51	51.03	33	18	Bilateral STN DBS	15	11.35
Zhou et al. [64]	11	43.8	9	2	Bilateral STN DBS	13.4	4.9
Lachenmayer et al. [32]	18	64.8	11	7	Unilateral and bilateral GPi DBS	5.5 and 10	16.2
Tsuboi et al. [65]	65	62.6	39	26	Bilateral GPi DBS	6 to 8	11.9
Hwang et al. [17]	40	59.5	22	18	Bilateral GPi DBS	5	11.4
Moro et al. [61]	16	54.4	11	5	Bilateral GPi DBS	5 to 6	14

Abbreviation: NR, not reported.

^∗^The study did not explicitly state unilateral or bilateral DBS methodology.

## Data Availability

All data for this review are available from the corresponding author upon reasonable request.

## References

[1] Zafar S., Yaddanapudi S. S. (2024). Parkinson Disease. *StatPearls [Internet]*.

[2] NIH National Institute on Aging (2022). Parkinson’s Disease: Causes, Symptoms, and Treatments.

[3] Schapira A. H. V., Emre M., Jenner P., Poewe W. (2009). Levodopa in the Treatment of Parkinson’s Disease. *European Journal of Neurology*.

[4] Magrinelli F., Picelli A., Tocco P. (2016). Pathophysiology of Motor Dysfunction in Parkinson’s Disease as the Rationale for Drug Treatment and Rehabilitation. *Parkinson’s Disease*.

[5] McGregor M. M., Nelson A. B. (2019). Circuit Mechanisms of Parkinson’s Disease. *Neuron*.

[6] Okun M. S. (2012). Deep-brain Stimulation for Parkinson’s Disease. *New England Journal of Medicine*.

[7] Dallapiazza R. F., De Vloo P., Fomenko A., Stoker T. B., Greenland J. C. (2018). Considerations for Patient and Target Selection in Deep Brain Stimulation Surgery for Parkinson’s Disease. *Parkinson’s Disease: Pathogenesis and Clinical Aspects*.

[8] Pycroft L., Stein J., Aziz T. (2018). Deep Brain Stimulation: An Overview of History, Methods, and Future Developments. *Brain and Neuroscience Advances*.

[9] Sharma V. D., Patel M., Miocinovic S. (2020). Surgical Treatment of Parkinson’s Disease: Devices and Lesion Approaches. *Neurotherapeutics*.

[10] Fang J. Y., Tolleson C. (2017). The Role of Deep Brain Stimulation in Parkinson’s Disease: An Overview and Update on New Developments. *Neuropsychiatric Disease and Treatment*.

[11] Spears C., Heston A. (2024). *Deep Brain Stimulation (DBS)*.

[12] Berney A., Vingerhoets F., Perrin A. (2002). Effect on Mood of Subthalamic DBS for Parkinson’s Disease: A Consecutive Series of 24 Patients. *Neurology*.

[13] Lachenmayer M. L., Mürset M., Antih N. (2021). Subthalamic and Pallidal Deep Brain Stimulation for Parkinson’s Disease—Meta-Analysis of Outcomes. *NPJ Parkinsons Disease*.

[14] Peng L., Fu J., Ming Y., Zeng S., He H., Chen L. (2018). The Long-Term Efficacy of STN vs GPi Deep Brain Stimulation for Parkinson Disease: A Meta-Analysis. *Medicine*.

[15] Zhang J., Li J., Chen F. (2021). STN Versus GPi Deep Brain Stimulation for Dyskinesia Improvement in Advanced Parkinson’s Disease: A Meta-Analysis of Randomized Controlled Trials. *Clinical Neurology and Neurosurgery*.

[16] Antonini A., Moro E., Godeiro C., Reichmann H. (2018). Medical and Surgical Management of Advanced Parkinson’s Disease. *Movement Disorders, Official Journal of the Movement Disorder Society*.

[17] Hwang Y. S., Jo S., Lee S. H. (2023). Long-term Motor Outcomes of Deep Brain Stimulation of the Globus Pallidus Interna in Parkinson’s Disease Patients: Five-Year Follow-Up. *Journal of the Neurological Sciences*.

[18] Jiang C., Wang J., Chen T., Li X., Cui Z. (2022). Short- and Long-Term Efficacy and Safety of Deep-Brain Stimulation in Parkinson’s Disease Patients Aged 75 Years and Older. *Brain Sciences*.

[19] Krause M., Fogel W., Heck A. (2001). Deep Brain Stimulation for the Treatment of Parkinson’s Disease: Subthalamic Nucleus Versus Globus Pallidus Internus. *Journal of Neurology, Neurosurgery & Psychiatry*.

[20] Rodriguez-Oroz M. C., Obeso J. A., Lang A. E. (2005). Bilateral Deep Brain Stimulation in Parkinson’s Disease: a Multicentre Study with 4 Years Follow-Up. *Brain*.

[21] Au K. L. K., Wong J. K., Tsuboi T. (2021). Globus Pallidus Internus (GPi) Deep Brain Stimulation for Parkinson’s Disease: Expert Review and Commentary. *Neurological Therapy*.

[22] Bove F., Mulas D., Cavallieri F. (2021). Long-term Outcomes (15 Years) After Subthalamic Nucleus Deep Brain Stimulation in Patients with Parkinson Disease. *Neurology*.

[23] Wong J. K., Viswanathan V. T., Nozile-Firth K. S. (2020). STN versus GPi Deep Brain Stimulation for Action and Rest Tremor in Parkinson’s Disease. *Frontiers in Human Neuroscience*.

[24] Odekerken V. J. J., Boel J. A., Schmand B. A. (2016). GPi vs STN Deep Brain Stimulation for Parkinson Disease: Three-Year Follow-Up. *Neurology*.

[25] DerSimonian R., Laird N. (2015). Meta-analysis in Clinical Trials Revisited. *Contemporary Clinical Trials*.

[26] R Core Team (2023). *R: A Language and Environment for Statistical Computing*.

[27] Viechtbauer W. (2010). Conducting Meta-Analyses in *R* With the Metafor Package. *Journal of Statistical Software*.

[28] Lezcano E., Gómez-Esteban J. C., Tijero B. (2016). Long-term Impact on Quality of Life of Subthalamic Nucleus Stimulation in Parkinson’s Disease. *Journal of Neurology*.

[29] Li J., Zhang Y., Li Y. (2015). Long-Term Follow-Up of Bilateral Subthalamic Nucleus Stimulation in Chinese Parkinson’s Disease Patients. *British Journal of Neurosurgery*.

[30] Okun M. S., Foote K. D. (2005). Subthalamic Nucleus vs Globus Pallidus Interna Deep Brain Stimulation, the Rematch: Will Pallidal Deep Brain Stimulation Make a Triumphant Return?. *Archives of Neurology*.

[31] Kleiner-Fisman G., Herzog J., Fisman D. N. (2006). Subthalamic Nucleus Deep Brain Stimulation: Summary and Meta-Analysis of Outcomes. *Movement Disorders*.

[32] Lachenmayer M. L., Bettschen C., Bernasconi C. (2019). Stimulation of the Globus Pallidus Internus in the Treatment of Parkinson’s Disease: Long-Term Results of a Monocentric Cohort. *Parkinsonism & Related Disorders*.

[33] Elgebaly A., Elfil M., Attia A., Magdy M., Negida A. (2018). Neuropsychological Performance Changes Following Subthalamic versus Pallidal Deep Brain Stimulation in Parkinson’s Disease: A Systematic Review and Metaanalysis. *CNS Spectrums*.

[34] Bucur M., Papagno C. (2023). Deep Brain Stimulation in Parkinson Disease: A Meta-Analysis of the Long-Term Neuropsychological Outcome. *Neuropsychology Review*.

[35] Wang Y., Li Y., Zhang X., Xie A. (2018). Apathy Following Bilateral Deep Brain Stimulation of Subthalamic Nucleus in Parkinson’s Disease: A Meta-Analysis. *Parkinson’s Disease*.

[36] Zoon T. J. C., Van Rooijen G., Balm G. M. F. C. (2021). Apathy Induced by Subthalamic Nucleus Deep Brain Stimulation in Parkinson’s Disease: A Meta‐analysis. *Movement Disorders*.

[37] Zhang S., Zi S., Xiong S., Peng H., Hu K., He H. (2022). Apathy Following Bilateral Deep Brain Stimulation of Subthalamic Nucleus and Globus Pallidus Internus in Parkinson’s Disease: A Meta-Analysis. *Parkinson’s Disease*.

[38] Alberts J. L., Voelcker-Rehage C., Hallahan K., Vitek M., Bamzai R., Vitek J. L. (2008). Bilateral Subthalamic Stimulation Impairs Cognitive-Motor Performance in Parkinson’s Disease Patients. *Brain*.

[39] Saint-Cyr J. A. (2000). Neuropsychological Consequences of Chronic Bilateral Stimulation of the Subthalamic Nucleus in Parkinson’s Disease. *Brain*.

[40] Lee D. J., Drummond N. M., Saha U. (2021). Acute Low Frequency Dorsal Subthalamic Nucleus Stimulation Improves Verbal Fluency in Parkinson’s Disease. *Brain Stimulation*.

[41] Bot M., Schuurman P. R., Odekerken V. J. J. (2018). Deep Brain Stimulation for Parkinson’s Disease: Defining the Optimal Location Within the Subthalamic Nucleus. *Journal of Neurology, Neurosurgery, and Psychiatry*.

[42] Cernera S., Eisinger R. S., Wong J. K. (2020). Long-Term Parkinson’s Disease Quality of Life After Staged DBS: STN vs GPi and First vs Second Lead. *NPJ Parkinsons Disease*.

[43] Park H. R., Im H. J., Park J. (2022). Long-term Outcomes of Bilateral Subthalamic Nucleus Deep Brain Stimulation for Patients with Parkinson’s Disease: 10 Years and Beyond. *Neurosurgery*.

[44] Thomsen B. L. C., Jensen S. R., Clausen A., Karlsborg M., Jespersen B., Løkkegaard A. (2020). Deep Brain Stimulation in Parkinson’s Disease: Still Effective After More Than 8 Years. *Movement Disorders in Clinical Practice*.

[45] Wider C., Pollo C., Bloch J., Burkhard P. R., Vingerhoets F. J. G. (2008). Long-term Outcome of 50 Consecutive Parkinson’s Disease Patients Treated With Subthalamic Deep Brain Stimulation. *Parkinsonism & Related Disorders*.

[46] Simonin C., Tir M., Devos D. (2009). Reduced Levodopa-Induced Complications after 5 Years of Subthalamic Stimulation in Parkinson’s Disease: a Second Honeymoon. *Journal of Neurolology*.

[47] Genovese D., Bove F., Rigon L. (2024). Long-term Safety and Efficacy of Frameless Subthalamic Deep Brain Stimulation in Parkinson’s Disease. *Neurological Sciences*.

[48] Shahidi G. A., Rohani M., Parvaresh M. (2017). Outcome of Subthalamic Nucleus Deep Brain Stimulation on Long-Term Motor Function of Patients With Advanced Parkinson Disease. *Iran Journal of Neurology*.

[49] Shin H. W., Kim M. S., Kim S. R., Jeon S. R., Chung S. J. (2020). Long-term Effects of Bilateral Subthalamic Deep Brain Stimulation on Postural Instability and Gait Difficulty in Patients with Parkinson’s Disease. *Journal of Molecular Diagnostics*.

[50] Jiang J. L., Chen S. Y., Tsai S. T., Ma Y. C., Wang J. H. (2023). Long-term Effects of Subthalamic Stimulation on Motor Symptoms and Quality of Life in Patients with Parkinson’s Disease. *Healthcare*.

[51] Jiang J. L., Chen S. Y., Hsieh T. C., Lee C. W., Lin S. H., Tsai S. T. (2015). Different Effectiveness of Subthalamic Deep Brain Stimulation in Parkinson’s Disease: A Comparative Cohort Study at 1 Year and 5 Years. *Journal of the Formosan Medical Association*.

[52] Romito L. M., Contarino M. F., Vanacore N., Bentivoglio A. R., Scerrati M., Albanese A. (2009). Replacement of Dopaminergic Medication With Subthalamic Nucleus Stimulation in Parkinson’s Disease: Long‐Term Observation. *Movement Disorders*.

[53] Jiang L. L., Chen W., Guo Q. (2021). Eight‐Year Follow‐up Outcome of Subthalamic Deep Brain Stimulation for Parkinson’s Disease: Maintenance of Therapeutic Efficacy with a Relatively Low Levodopa Dosage and Stimulation Intensity. *CNS Neuroscience and Therapeutics*.

[54] Jiang L. L., Liu J. L., Fu X. L. (2015). Long-term Efficacy of Subthalamic Nucleus Deep Brain Stimulation in Parkinson’s Disease: A 5-Year Follow-Up Study in China. *Chinese Medical Journal*.

[55] Janssen M. L. F., Duits A. A., Toura A. M. (2014). Subthalamic Nucleus High-Frequency Stimulation for Advanced Parkinson’s Disease: Motor and Neuropsychological Outcome after 10 Years. *Stereotactic and Functional Neurosurgery*.

[56] Contarino M. F., Daniele A., Sibilia A. H. (2006). Cognitive Outcome 5 Years after Bilateral Chronic Stimulation of Subthalamic Nucleus in Patients With Parkinson’s Disease. *Journal of Neurology, Neurosurgery & Psychiatry*.

[57] Rizzone M. G., Fasano A., Daniele A. (2014). Long-term Outcome of Subthalamic Nucleus DBS in Parkinson’s Disease: From the Advanced Phase towards the Late Stage of the Disease?. *Parkinsonism & Related Disorders*.

[58] Kim R., Kim H. J., Shin C. (2019). Long-Term Effect of Subthalamic Nucleus Deep Brain Stimulation on Freezing of Gait in Parkinson’s Disease. *Journal of Neurosurgery*.

[59] Bjerknes S., Toft M., Brandt R. (2021). Subthalamic Nucleus Stimulation in Parkinson’s Disease: 5‐Year Extension Study of a Randomized Trial. *Movement Disorders Clinical Practice*.

[60] Guo S., Li J., Zhang Y., Li Y., Zhuang P. (2021). Optimal Target Localisation and Eight-Year Outcome for Subthalamic Stimulation in Patients with Parkinson’s Disease. *British Journal of Neurosurgery*.

[61] Moro E., Lozano A. M., Pollak P. (2010). Long‐Term Results of a Multicenter Study on Subthalamic and Pallidal Stimulation in Parkinson’s Disease. *Movement Disorders*.

[62] Schupbach W. M. M., Chastan N., Welter H. L. (2005). Stimulation of the Subthalamic Nucleus in Parkinson’s Disease: A 5 Year Follow Up. *Journal of Neurology, Neurosurgery & Psychiatry*.

[63] de Noordhout A. M., Mouchamps M., Remacle J. M., Delstanche S., Bonhomme V., Gonce M. (2022). Subthalamic Deep Brain Stimulation Versus Best Medical Treatment: A 12-Year Follow-Up. *Acta Neurologica Belgica*.

[64] Zhou H., Wang L., Zhang C. (2019). Acute Effects of Subthalamic Deep Brain Stimulation on Motor Outcomes in Parkinson’s Disease; 13 Year Follow Up. *Fronters in Neurolology*.

[65] Tsuboi T., Lemos Melo Lobo Jofili Lopes J., Moore K. (2021). Long-Term Clinical Outcomes of Bilateral GPi Deep Brain Stimulation in Advanced Parkinson’s Disease: 5 Years and Beyond. *Journal of Neurosurgery*.

